# Association of COVID-19 and Lung Cancer: Short-Term and Long-Term Interactions

**DOI:** 10.3390/cancers16020304

**Published:** 2024-01-11

**Authors:** Ying-Long Peng, Zi-Yan Wang, Ri-Wei Zhong, Shi-Qi Mei, Jia-Qi Liu, Li-Bo Tang, Zhi Guo, Zi-Rui Ren, Lv Wu, Yu Deng, Zhi-Hong Chen, Qing Zhou, Chong-Rui Xu

**Affiliations:** 1 School of Medicine, South China University of Technology, Guangzhou 510006, Chinazrw1106@163.com (R.-W.Z.);; 2 Guangdong Lung Cancer Institute, Guangdong Provincial People’s Hospital (Guangdong Academy of Medical Sciences), Southern Medical University, Guangzhou 510180, Chinaliujiaqi3010@163.com (J.-Q.L.); guozhii123456@163.com (Z.G.);; 3 The First Clinical School, Guangzhou Medical University, Guangzhou 510120, China

**Keywords:** COVID-19, lung cancer, PFS, SARS-CoV-2, targeted therapy

## Abstract

**Simple Summary:**

Since 2019, COVID-19 has had major effects around the world. Lung cancer and COVID-19 are both diseases of the respiratory system, and it is interesting to look into how they affect each other. However, very few studies look at how COVID-19 and lung cancer together. The point of this study was to look into the short- and long-term relationships between COVID-19 and lung cancer in order to find out how COVID-19 affects the outcome of lung cancer patients. We discovered that lung cancer patients infected with COVID-19 may have their disease progress more quickly. This suggests lung cancer patients infected with COVID-19 may need to have their tumor treatment evaluated more often so their treatment plans can be changed as needed.

**Abstract:**

**Background**: COVID-19 has been ravaging the globe for more than three years. Due to systemic immunosuppression of anti-tumor therapy, application of chemotherapy and adverse effects of surgery, the short- and long-term prognosis of cancer patients to COVID-19 are of significant concern. **Method**: This research included three parts of data. The first part of the data came from the public database that covered Veneto residents. The second part of the data included participants in Guangzhou. The third part of the data was used for MR analysis. We assessed the associations by logistic, linear or Cox regression when appropriate. **Result**: Lung cancer patients with COVID-19 had shorter progression-free survival (PFS) after COVID-19 (Model II: HR: 3.28, 95% CI: 1.6~6.72; Model III: HR: 3.39, 95% CI: 1.45~7.95), compared with lung cancer patients without COVID-19. Targeted therapy patients recovered from SARS-CoV-2 infection more quickly (Model I: β: −0.58, 95% CI: −0.75~−0.41; Model II: β: −0.59, 95% CI: −0.76~−0.41; Model III: β: −0.57; 95% CI: −0.75~−0.40). **Conclusions**: PFS in lung cancer patients is shortened by COVID-19. The outcome of COVID-19 in lung cancer patients was not significantly different from that of the healthy population. In lung cancer patients, targeted therapy patients had a better outcome of COVID-19, while chemotherapy patients had the worst.

## 1. Introduction

The severe acute respiratory syndrome coronavirus 2 (SARS-CoV-2) was the cause of the coronavirus disease 2019 (COVID-19). Since 31 December 2019, COVID-19 has ravaged the globe for over three years. Currently, COVID-19 is prevalent and has a pervasive influence on individuals globally. Due to the Chinese government’s aggressive management control of COVID-19, China had a low number of COVID-19 case reports. However, with the change in management measures, China may face a huge challenge from COVID-19 under the new measures.

The susceptibility of cancer patients to infection by SARS-CoV-2 and the subsequent prognosis has garnered significant interest among the academic community. Rugge et al. found a lower higher proportion of hospitalization (56.6% vs. 34.4%) and death (14.7% vs. 4.5%) for COVID-19 in cancer patients [1]. A meta-analysis suggested both lung cancer (HR: 2.00, 95% CI: 1.52~2.63) and non-lung cancer patients (HR: 1.91, 95% CI: 1.53~2.39) had a higher risk of death from COVID-19 [2]. Dai et al. reported cancer patients had worse COVID-19 outcomes (death: OR: 2.84, 95% CI: 1.59~5.08; severe symptoms: OR: 2.79, 95% CI: 1.74~4.41) [3]. These studies have shown that cancer patients who had COVID-19 exhibited a worse outcome compared to healthy individuals. Wang et al. reported lung cancer patients had a higher possibility of SARS-CoV-2 infection (OR: 7.14; 95% CI: 6.91~7.39) [4]; Yu et al. suggested SARS-CoV-2 high infectivity in cancer patients was associated with multiple medical appointments and hospitalizations [5]. Overall, current research on cancer and COVID-19 is incomplete and has only examined short-term effects.

Different anti-cancer treatment strategies have varying effects on patients. Current research suggests that chemotherapy made patients susceptible to infections, while targeted therapy and chemotherapy had small impacts on infections [6,7,8]. Since both COVID-19 and lung cancer are respiratory diseases, it is important to focus on the susceptibility and prognosis of COVID-19 in lung cancer patients treated with different medications. Additionally, infection by many pathogens is thought to play an important role in cancer development; for example, Helicobacter pylori is considered a risk factor for the development of gastric cancer [9]. The potential prognostic implications of SARS-CoV-2, a novel pathogen with a primary affinity for the respiratory system, on patients with lung cancer remain uncertain.

In this study, we involved both healthy individuals and cancer patients to investigate potential disparities in susceptibility to COVID-19. We also examined the prognosis of COVID-19 in both healthy individuals and cancer patients. Within the subgroup of lung cancer patients, we conducted comparisons to identify variances in susceptibility and COVID-19 prognosis among individuals undergoing different treatment strategies. Furthermore, we conducted an analysis to assess the progression-free survival (PFS) of lung cancer patients who contracted COVID-19, aiming to ascertain the influence of COVID-19 on oncology treatment outcomes.

## 2. Materials and Methods

### 2.1. Data Sources and Study Population

In order to improve the accuracy of our study and make the findings applicable to a wider range of scenarios, we included participants from two sources. The first group of individuals came from a public database authored by Rugge et al. [10], including Veneto residents who tested for SARS-CoV-2 infection between 22 February and 1 April 2020. The second part of individuals was collected from Guangdong Provincial People’s Hospital between 26 January and 28 March after China’s coronavirus disease 2019 (COVID-19) pandemic. The two parts of the data were analyzed, respectively. Overall, the two sources of data were used to make the results more accurate. The accuracy of the results could be demonstrated if two-part studies from different times, regions, ethnicities, and SARS-CoV-2 strains showed similar findings. All participants that were recruited provided their informed consent.

Wang et al. reported the genetic data of lung cancer (IEU GWAS ID: ieu-a-966) [11]; COVID-19 genetic data were acquired from the COVID-19 Host Genetics Initiative [12]. COVID-19 (hospitalized vs. population) RELEASE 5 and COVID-19 (very severe respiratory confirmed vs. population) RELEASE 5 were selected. Dyspnea, respiratory rate ≤ 30/min, SpO_2_ ≤ 93%, PaO_2_/FiO_2_ < 300 mmHg, or greater than 50% lung field infiltration constituted severe COVID-19 [13]. Guangdong Provincial People’s Hospital gave its approval to this research, which was carried out in accordance with the Helsinki Declaration.

### 2.2. Diagnosis of SARS-CoV-2 Infection and Prognosis

For individuals from the first source, the SARS-CoV-2 infection was sought and confirmed by real-time Polymerase Chain Reaction (PCR) with reverse transcription and next-generation sequencing [1]. In the second source, SARS-CoV-2 infection was identified by PCR or antibody test. Symptom duration or recovery time referred to the length of time between SARS-CoV-2 infection diagnosis and the patient’s self-perceived disappearance or recovery; both variables were continuous. Medical attendance and hospitalization were categorical variables that indicated whether a patient received medical assistance or was hospitalized for SARS-CoV-2 infection. Different symptoms of COVID-19 were categorical variables. As a result of SARS-CoV-2 infection, patients with lung cancer were unable to receive anti-cancer treatment for a period of time. We defined the interruption of lung cancer treatment for less than one week as having no effect on cancer treatment and otherwise as having an impact.

### 2.3. Cancer Diagnosis, Medication Strategies and Prognosis

For the first source individuals, the patient’s cancer types were obtained from public databases or pathology reports; the patient’s cancer type was classified into participants without cancer, patients with non-lung cancer, and patients with lung cancer. For individuals from the second source, all involved lung cancer patients received surgery or biopsied with a pathological diagnosis of lung cancer. Patients’ cancer types in the second part were divided into patients without cancer and patients with lung cancer. Lung cancer medication referred to the medication options currently used by patients, which were divided into three groups: targeted therapy group, immunotherapy group and chemotherapy group. All PFS analyses measured the time from the beginning of current lung cancer treatment to the time of cancer progress or last follow-up. Specifically, we calculated the PFS after COVID-19 (PFSC), which referred to the time from SARS-CoV-2 infection to cancer progress or last follow-up. For patients not infected with SARS-CoV-2, we used the mean time of patients infected with SARS-CoV-2 as a proxy. For patients who did not have an anti-cancer treatment before SARS-CoV-2 infection, PFSC was measured from the time of initiation of treatment.

### 2.4. Covariates

Due to the limitation of public databases, only age and sex were addressed as covariates in the first part of individuals. For healthy individuals from the second source, we collected information about age, sex, vaccination status, smoking status, and underlying disease as covariates. For lung cancer patients, age, sex, lung cancer pathology type, SARS-CoV-2 vaccine status, COVID-19 treatment options, chest radiotherapy, underlying diseases, smoking status, smoking index and performance status (PS) were used as covariates. Lung cancer pathology types were divided into four categories based on pathological diagnosis by puncture or surgery: squamous carcinoma, adenocarcinoma, other non-small cell lung cancer (NSCLC), and small cell lung cancer (SCLC). We categorized patients based on the number of COVID-19 vaccinations received: unvaccinated, one dose received, two doses received, and three or four doses received. COVID-19 treatment options included severe treatment: non-steroidal anti-inflammatory drugs (NSAIDs), antibiotics, Chinese medicine, oxygen therapy, glucocorticoid, Paxlovid, and other treatment and untreated. Smoking status included current smoker, former smoker and never smoker. In a partial regression, the smoking index values were too large to visualize the results of the regression. In order to express the results of regression more visually, the smoking index values were narrowed to 1% for static. Lung cancer treatment strategies were categorized according to the number of lines into first line, second line and ≥third line.

### 2.5. Statistical Analysis

In this study, the statistical program R-4.0.2 (The R Foundation) and Free Statistics software version 1.7.1 were used to analyze. Means, standard errors, medians, percentages, and frequencies were used to describe the baseline characteristics when appropriate. The odds ratio (OR), regression coefficients (β) and hazard ratio (HR) were used to estimate the association between lung cancer and COVID-19 in logistic regression, linear regression, and Cox regression. A 95% confidence interval (CI) was also used to evaluate the strength of the association. Normally distributed continuous variables were compared by a *t*-test or analysis of variance. Non-parametric tests were applied to compare continuous data that did not meet a normal distribution. Categorical variables were analyzed by the chi-square test or Fisher test. In linear regression, variables that do not conform to a normal distribution are treated using a square transformation. Interaction effect analysis was used in subgroup analysis. The threshold for statistical significance was set at *p* < 0.05.

Mendelian randomization (MR) analysis was also used to evaluate the difference in SARS-CoV-2 infection and prognosis between lung cancer patients and the healthy population. In the MR analysis, lung cancer was used as an exposure factor, while hospitalization and severe COVID-19 were the outcomes. Eleven single-nucleotide polymorphisms (SNPs) robustly correlated with lung cancer (*p* < 5 × 10^−8^) were extracted as instrumental variables (IVs). Using the clump data method, we eliminated the strongly correlated variants with r^2^ < 0.1 within 10 Mb (kb > 10,000) to eliminate valid SNPs. For the MR analysis, the inverse variance weighted (IVW) method was mostly used. The heterogeneity test, horizontal multiple validity test and leave-one-out analysis were used to assess the validity and reliability of the MR analysis.

## 3. Results

### 3.1. Baseline Characteristics of Participants

**Figure 1:**
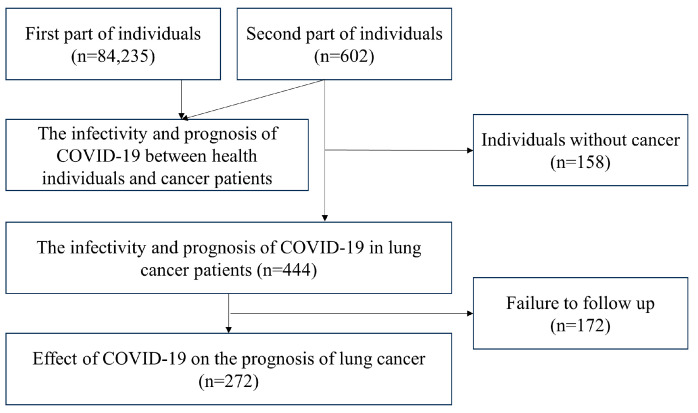
The flowchart of the study design.

This study involved 84,235 participants and 602 participants from the database and Guangdong Provincial People’s Hospital, respectively. The flowchart (Figure 1) shows the details of the study design. The baseline characteristics of participants from the database by Rugge et al. are shown in Supplementary Table S1. Supplementary Tables S2–S4 included baseline characteristics of participants from Guangdong Provincial People’s Hospital.

### 3.2. The Infectivity and Prognosis of COVID-19 between Healthy Individuals and Cancer Patients

The univariate analysis indicated that sex and cancer types were associated with SARS-CoV-2 infection in both sources (Supplementary Tables S5 and S6). Symptom duration was not associated with any variates (Supplementary Table S7). Recovery time of COVID-19 was associated with age and vaccination (Supplementary Table S8).

After adjusting for covariates, patients with lung cancer from two sources both suggested a lower possibility of SARS-CoV-2 infection (first part: OR: 0.61; 95% CI: 0.39~0.97; second part: OR: 0.41; 95% CI: 0.18~0.94) (Table 1). Both data sets revealed similar findings, indicating a stable connection between lung cancer and persistent SARS-CoV-2 infection. The multiple linear regression showed symptom duration was not associated with lung cancer (β: −0.19; 95%CI: −0.56~0.18) and similar recovery time (β: −0.14; 95%CI: −0.34~0.06) (Supplementary Tables S9 and S10).

**Figure 2:**
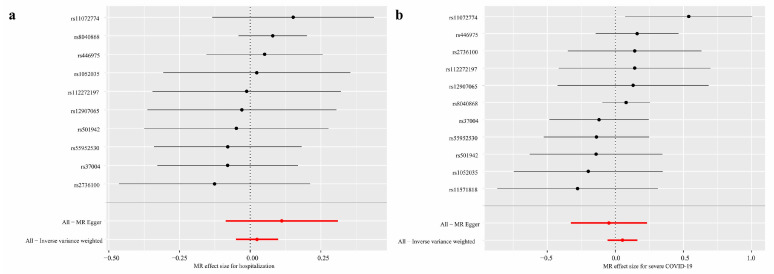
The MR analysis of the association between lung cancer and COVID-19 outcomes. (**a**) The MR analysis of the association between lung cancer and hospitalization for COVID-19; (**b**) the MR of association between lung cancer and severe COVID-19.

Ten and eleven valid SNPs were extracted for hospitalization and severe COVID-19, respectively. The MR analysis showed there was no correlation between lung cancer and hospitalization after COVID-19 (OR_IVW_: 1.02; 95% CI: 0.95~1.10), similar in severe COVID-19 (OR_IVW_: 1.05; 95% CI: 0.94~1.18) (Figure 2). The MR sensitivity analysis and heterogeneity test indicated that both the Cochran’s Q statistic and the MR Egger regression intercept did not reach statistical significance (*p* > 0.05), suggesting the lack of heterogeneity and horizontal pleiotropy (Supplementary Figures S1 and S2, Supplementary Table S11).

### 3.3. The Infectivity and Prognosis of COVID-19 in Different Medication Lung Cancer Patients

The univariate analysis between lung cancer medication and SARS-CoV-2 infection is presented in Supplementary Table S12. The multiple logistic regression suggested different medications for lung cancer were not associated with SARS-CoV-2 infection (Supplementary Table S13).

Both univariate and multiple logistic regression were performed to assess the possibility of different symptoms appearing after COVID-19. The findings of the analysis indicated that targeted therapy had a higher propensity for inducing symptoms such as stuffy nose (OR: 2.37; 95% CI: 1.12~5.01), constipation (OR: 3.91; 95% CI: 1.37~11.17), and tired (OR: 2.32; 95% CI: 1.25~4.33) after COVID-19. Patients with immunotherapy had an increased likelihood of experiencing stuffy nose (OR: 2.91; 95% CI: 1.08~7.82) (Supplementary Table S14). There was no relationship between different lung cancer treatments and symptoms duration of COVID-19 (Supplementary Tables S15 and S16).

Medical attendance and hospitalization were also analyzed by univariate and multiple logistic regression (Supplementary Tables S17–S20). In univariate analysis, we found patients with higher PS had a higher possibility of receiving medical treatment; the older patients also had a higher possibility of receiving hospital treatment. Compared with chemotherapy patients, immunotherapy patients had a lower possibility of hospitalization for COVID-19 after adjusting for covariates (Model II: OR: 0.04, 95% CI: 0.00~0.68; Model III: OR: 0.01, 95% CI: 0.00~0.80).

After COVID-19, the recovery time was connected with age and PS in univariate analysis (Supplementary Table S21). In multiple linear regression, targeted therapy patients had a shorter time after COVID-19 in all models compared with chemotherapy patients (Model I: β: −0.58, 95% CI: −0.75~−0.41; Model II: β: −0.59, 95% CI: −0.76~−0.41; Model III: β: −0.57; 95% CI: −0.75~−0.40). There was no difference in COVID-19 recovery time between immunotherapy and chemotherapy (Table 2).

The anti-cancer therapy profile of lung cancer patients during COVID-19 was also affected. Patients with targeted therapy showed a lower possibility of discontinuing anti-cancer therapy compared with chemotherapy patients (Model I: OR: 0.12; 95% CI: 0.07~0.22; Model II: OR: 0.14; 95% CI: 0.07~0.27; Model III: OR: 0.12; 95% CI: 0.06~0.25). There was no difference in the cessation of anti-cancer profile between chemotherapy and immunotherapy patients (Table 3).

### 3.4. Effect of COVID-19 on the Prognosis of Lung Cancer

**Figure 3:**
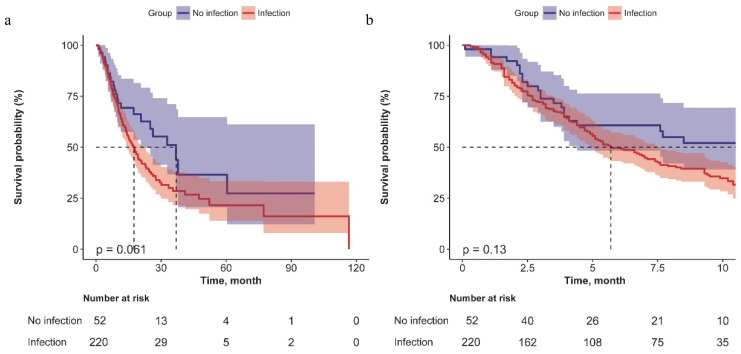
Kaplan–Meier Curves for PFS (**a**) and PFSC (**b**) of lung cancer based on COVID-19.

The Kaplan–Meier survival curve demonstrated that lung cancer patients with a history of COVID-19 had shorter PFS and PFSC, albeit these findings were not statistically significant (Figure 3). Cox regression suggested lung cancer patients with COVID-19 had shorter PFS (Model II: HR: 3.28, 95% CI: 1.6~6.72; Model III: HR: 3.39, 95% CI: 1.45~7.95) and PFSC (Model II: HR: 2.83, 95% CI: 1.39~5.76; Model III: HR: 3.84, 95% CI: 1.67~8.84) than patients without COVID-19, after adjusting for covariates (Table 4).

Subgroup analysis was performed according to lung cancer treatment (Supplementary Tables S22 and S23). In the univariate model, the PFS (HR: 1.48; 95% CI: 0.84~2.61) and PFSC (HR: 1.34; 95% CI: 0.76~2.37) of targeted therapy patients with COVID-19 were shorter, compared with patients without COVID-19, though these results were not statistically significant. Compared with patients without COVID-19, the PFS and PFSC were shorter for targeted therapy patients with COVID-19 after adjusting for covariates (PFS: HR: 5.96; 95% CI: 1.91~18.58; PFSC: HR: 5.2, 95% CI: 1.65~16.38). As there were no instances of disease progression among the immunotherapy patients without COVID-19 at the time of the statistical analysis, the statistical analyses conducted on these individuals provided findings that lacked statistical significance. Notably, the relationships between lung cancer treatment and PFS or PFSC in subgroup analysis were not statistically significant (all *p* for interaction > 0.05).

## 4. Discussion

This is the first article to examine the interaction between COVID-19 and lung cancer. We found lung cancer patients had shorter PFS after COVID-19. Additionally, lung cancer patients had a lower possibility of SARS-CoV-2 infection. In this study, there was no difference between lung cancer patients and the healthy population in the prognosis of COVID-19. In lung cancer patients, targeted therapy patients had the best prognosis after COVID-19, while those treated with chemotherapy had the worst prognosis. This study collected data from three sources and adjusted covariates by multiple regression to reduce bias.

In general, many studies reported that cancer patients had a poor prognosis when infected by COVID-19 but only a few studies examined the association between SARS-CoV-2 infection and cancer. Two studies reported that cancer patients had higher infectivity to SARS-CoV-2 [4,5]: one of these studies noted that cancer patients’ high infectivity to SARS-CoV-2 was associated with multiple medical appointments and hospitalizations. Above all, SARS-CoV-2 susceptibility in cancer patients remained unclear. In this study, we included patients at the peak of the COVID-19 outbreak in China. Prior to this period, cancer patients were unable to visit the hospital on a regular basis due to policies that reduced interference with medical appointments and hospitalizations. Due to the limitations imposed by the prevailing policy throughout the study period, our research was able to mitigate the impact of healthcare-acquired infections to a certain degree. 

To our knowledge, we first proposed that lung cancer patients had a lower possibility of SARS-CoV-2 infection compared with participants without cancer. Our findings seem to be contrary to our usual perception. We proposed some potential hypotheses: 1. Erdem et al. showed that cancer patients’ education seemed to increase their protective awareness [14]. Increased protective awareness may decrease the possibility of SARS-CoV-2 infection. 2. Angiotensin-converting enzyme 2 (ACE2) is the principal route by which SARS-CoV-2 penetrates human cells [15]. Current studies suggest the expression of ACE2 was decreased in lung cancer patients [16,17]. The reduced ACE2 expression in lung cancer patients may lead to the more difficult entry of SARS-CoV-2 into the tissues and cells of lung cancer patients, resulting in low SARS-CoV-2 infection rates in lung cancer patients. Numerous studies have examined the outcomes and prognosis of COVID-19, which found cancer patients with COVID-19 have a relatively dismal prognosis [2,3,18]. Our study suggested that there is no difference in the prognoses of lung cancer patients with COVID-19 compared to the healthy population. The main factors used in this study to measure prognosis were recovery time and duration of symptoms. Most previous studies have used hospitalization and severe COVID-19 as prognoses. This difference may lead to different results. In order to reduce bias due to differences in outcome indicators, we performed an MR analysis. It was shown that there was no genetic correlation between lung cancer and heightened susceptibility to hospitalization or severe manifestations of COVID-19, as evidenced by the analysis of an extensive genome-wide association study. Among the different medication options for lung cancer patients, we found targeted therapy had better outcomes, while chemotherapy had a worse prognosis.

Programmed cell death ligand 1 (PD-L1) is expressed in several cancer cells and has the ability to selectively attach to programmed cell death protein 1 (PD-1) on tumor antigen-specific T cells. This interaction hinders the ability of T cells to eliminate tumor cells [19]. ICIs such as PD-1/PD-L1 monoclonal antibodies can inhibit the binding of PD-1 and PD-L1, thereby promoting T-cell activation and release of inflammatory cytokines [20]. This response enables T cells to exert anti-cancer effects in cancerous tissues. In normal tissues, such a response appears to enhance the level of immune response to pathogenic infection, reduce the incidence of infection and improve the prognosis of infection. This hypothesis is consistent with our study’s finding that immunotherapy patients have a diminished risk of hospitalization.

Nonetheless, some studies suggest that hyperinflammatory dysregulated immunity due to ICIs may increase the risk of infection [21]. The study conducted by Robilotti et al. showed a positive correlation between immunotherapy treatment and an increased likelihood of hospitalization for COVID-19 [22], which was different from our results. Fujita et al. reported that the majority of infections in ICI users are bacterial, while viral infections are less severe [23]. Considering the different SARS-CoV-2 genotypes of this study and Robilotti’s study, we hypothesized that a high inflammatory immune status would control the infection for less pathogenic strains. For more pathogenic strains, a high inflammatory immune status would not effectively control the infection and could develop into a cytokine storm. Immune-related adverse events (irAEs) were associated with ICIs in immunotherapy [24]. Glucocorticoids were used to treat irAEs, leading to an increased chance of infection; however, Fujita et al. reported no difference in the use of corticosteroids or immunosuppressive agents between patients with and without infections [23]. Above all, the relationship between immunotherapy and infection and immune response in patients is not well understood, and future research is required to elucidate this relationship and the distinctions in immunotherapy for infections caused by different pathogens.

Targeted therapy patients had shorter recovery time after COVID-19. In addition, lung cancer patients using targeted therapy showed lower possibility of discontinuing anti-cancer therapy. Targeted therapies target specific molecular targets of cancer cells to exert anti-tumor effects; therefore, targeted therapies often do not have a direct effect on the immune function of the body [25]. Common adverse events of targeted therapy include rash, hypertension, hypothyroidism, proteinuria, and an increased risk for venous and gastrointestinal toxicity [26,27,28]. There is insufficient evidence that targeted therapy directly affects immune function; therefore, we consider that patients on targeted therapy may have a better immune response to infection compared to cancer patients who received immunotherapy and chemotherapy, which may contribute to their quicker recovery. In addition, since most of the targeted drugs are tablets, which can be taken at home, immune and chemotherapeutic drugs are often injections, which must be administered in the hospital. We hypothesize that this factor may also contribute to the lower possibility of discontinuing anti-cancer therapy in targeted therapy patients and the stronger immune response to infection in targeted therapy patients.

Infection is thought to have a significant impact on the development of some cancers. Examples are human papillomavirus (HPV) for cervical cancer and Epstein-Barr virus for nasopharyngeal cancer [29]. HPV-18 has been shown to be a prognostic risk factor for cervical cancer [30]. In the case of gastric cancer, Helicobacter pylori has been considered a protective factor for the prognosis of gastric cancer [31]. The occurrence of infections during the development of lung cancer may increase the incidence of lung cancer and lead to metastasis and recurrence of lung cancer [32]. SARS-CoV-2 infection leads to lung inflammation [33], and inflammation causes DNA damage by inducing cell proliferation and increasing reactive oxygen species [34], which may increase the incidence of lung cancer. Targeted therapy is one of the most commonly used treatments in patients with advanced lung cancer [35]. It is currently believed that one of the causes of resistance to targeted therapy is the development of drug-resistant mutations [36]. Lung inflammation caused by COVID-19 may lead to drug resistance by increasing the probability of DNA damage and the probability of mutation development, which in turn leads to drug resistance. The reported mechanism aligns with our research findings, as indicated by the subgroup analyses conducted in our study. These analyses revealed that patients receiving targeted therapy had poorer PFS and PFSC following COVID-19 infection. Conversely, no such association was identified among patients receiving immunotherapy and chemotherapy.

This study has some limitations. Firstly, we included data from two sources in our observational study, and there were differences in covariates between the two parts of the data that could lead to bias. The two sections of the data consistently provide similar findings despite their variations. Their mutual validation reinforces the credibility of our conclusions. Secondly, we did not choose hospitalization, severe COVID-19, and death, which have been commonly used in previous studies, as our prognostic outcome indicators. We performed hospitalization and severe COVID-19 in MR analysis, enabling our study to accurately respond to the COVID-19 prognosis of lung cancer patients from more perspectives. Thirdly, in the subgroup analysis, the fraction of patients who did not have COVID-19 and received immunotherapy showed no disease progression. Consequently, subgroup regression failed to provide statistically significant findings for this particular grouping. Subsequent investigations should delve further into the impact of COVID-19 on PFS in lung cancer patients undergoing immunotherapy.

## 5. Conclusions

Overall, we found lung cancer patients have shorter PFS after COVID-19. This finding suggests we should assess cancer status more frequently in lung cancer patients with a history of COVID-19 to develop an appropriate treatment plan. Additionally, lung cancer patients may have a lower risk of SARS-CoV-2 infection and similar prognoses after infection compared to the healthy population. Among lung cancer patients, patients with targeted therapy have a better prognosis for COVID-19. The worse prognosis of lung cancer patients in the context of COVID-19 is a significant matter that warrants more investigation and debate in the future. Future studies are necessary to further examine the relationship between acute infections and cancers. Furthermore, it is necessary to explore the different mechanisms played by ICIs in the infection of different pathogens.

## Figures and Tables

**Table 1:** The relationship between SARS-CoV-2 infection and cancer types.

Cancer Types	Model I		Model II	
	OR (95% CI)	*p*	OR (95% CI)	*p*
**First part of data**				
Without cancer	1 (reference)		1 (reference)	
Non-lung cancer	1.49 (1.37~1.62)	<0.001	0.98 (0.90~1.07)	0.613
Lung cancer	1.1 (0.70~1.74)	0.677	0.61 (0.39~0.97)	0.036
**Second part of data**				
Without cancer	1 (reference)		1 (reference)	
Lung cancer	0.52 (0.28~0.95)	0.034	0.41 (0.18~0.94)	0.036

Model I: Unadjusted; Model II in the first part: Adjusted for sex and age. Model II in the second part: Adjusted for sex, age, pathology, vaccination, chest radiotherapy, underlying disease, smoking status, PS and smoking index.

**Table 2:** The relationship between recovery time of COVID-19 and lung cancer treatment.

Treatment	Model I		Model II		Model III	
	β (95% CI)	*p*	β (95% CI)	*p*	β (95% CI)	*p*
Chemotherapy	0 (reference)		0 (reference)		0 (reference)	
Immunotherapy	−0.17 (−0.41~0.06)	0.145	−0.05 (−0.3~0.2)	0.712	−0.01 (−0.25~0.23)	0.932
Targeted therapy	−0.58 (−0.75~−0.41)	<0.001	−0.59 (−0.76~−0.41)	<0.001	−0.57 (−0.75~−0.4)	<0.001

Model I: Unadjusted; Model II: Adjusted for sex, age, pathology, vaccination, chest radiotherapy, underlying disease, smoking status, PS and smoking index; Model III: Adjusted for the variables in Model II plus COVID-19 treatment options.

**Table 3:** The relationship between anti-cancer for COVID-19 and lung cancer treatment.

Treatment	Model I		Model II		Model III	
	OR (95% CI)	*p*	OR (95% CI)	*p*	OR (95% CI)	*p*
Chemotherapy	1 (reference)		1 (reference)		1 (reference)	
Immunotherapy	1.19 (0.58~2.43)	0.627	0.84 (0.35~2.06)	0.71	0.94 (0.37~2.42)	0.906
Targeted therapy	0.12 (0.07~0.22)	<0.001	0.14 (0.07~0.27)	<0.001	0.12 (0.06~0.25)	<0.001

Model I: Unadjusted; Model II: Adjusted for sex, age, pathology, vaccination, chest radiotherapy, underlying disease, smoking status, PS and smoking index; Model III: Adjusted for the variables in Model II plus COVID-19 treatment options.

**Table 4:** Association of lung cancer prognosis and COVID-19.

COVID-19	Model I		Model II		Model III	
	HR (95% CI)	*p*	HR (95% CI)	*p*	HR (95% CI)	*p*
**PFS**						
No infection	1 (reference)		1 (reference)		1 (reference)	
Infection	1.52 (0.98~2.38)	0.063	3.28 (1.6~6.72)	0.001	3.39 (1.45~7.95)	0.005
**PFSC**						
No infection	1 (reference)		1 (reference)		1 (reference)	
Infection	1.41 (0.9~2.2)	0.13	2.83 (1.39~5.76)	0.004	3.84 (1.67~8.84)	0.002

Model I: Unadjusted; Model II: Adjusted for sex, age, pathology, vaccination, lung cancer treatment, treatment line, chest radiotherapy, anti-cancer therapy profile, underlying disease, smoking status, PS and smoking index; Model III: Adjusted for the variables in Model II plus COVID-19 treatment options. PFS: progression-free survival; PFSC: PFS after COVID-19.

## Data Availability

The detailed data of this study are available from the corresponding authors.

## Supplementary Materials

The following supporting information can be downloaded at: https://www.mdpi.com/article/10.3390/cancers16020304/s1. Figure S1: The leave one out sensitivity test for lung cancer and hospitalization after COVID-19; Figure S2: The leave one out sensitivity test for lung cancer and severe COVID-19; Table S1. Baseline characteristics of participants from database; Table S2. Baseline characteristics of participants from Guangdong Provincial People’s Hospital; Table S3. Baseline characteristics of lung cancer patients from Guangdong Provincial People’s Hospital; Table S4. Baseline characteristics of follow-up lung cancer patients from Guangdong Provincial People’s Hospital; Table S5. Association of variates and SARS-CoV-2 infection in participants from database; Table S6. Association of variates and SARS-CoV-2 infection in participants from Guangdong Provincial People’s Hospital; Table S7. Association of variates and symptoms duration of COVID-19; Table S8. Association of variates and recovery time of COVID-19; Table S9. Association between cancer types and symptoms duration of COVID-19; Table S10. Association between cancer types and recovery time of COVID-19; Table S11. Heterogeneity test and horizontal pleiotropy test of MR analysis; Table S12. Association of variates and SARS-CoV-2 infection in lung cancer patients; Table S13. Association of medication and SARS-CoV-2 infection in lung cancer patients; Table S14. Association of medication and COVID-19 symptoms in lung cancer patients; Table S15. Association of covariates and symptoms duration of COVID-19 in lung cancer patients; Table S16. Association between lung cancer medication and symptoms duration of COVID-19; Table S17. Association of covariates and medical attendance of COVID-19 in lung cancer patients; Table S18. Association of covariates and hospitalization of COVID-19 in lung cancer patients; Table S19. Association between lung cancer medication and medical attendance of COVID-19; Table S20. Association between lung cancer medication and hospitalization of COVID-19; Table S21. Association of covariates and recovery time of COVID-19 in lung cancer patients; Table S22. Association of PFS and COVID-19 in different subgroups; Table S23. Association of PFSC and COVID-19 in different subgroups.
