# Supplementary material for: Association of COVID-19 and Lung Cancer: Short-Term and Long-Term Interactions
Source: Cancers (Basel). 2024 Jan 11;16(2):304. doi: 10.3390/cancers16020304 (PMC10813989; doi:10.3390/cancers16020304)
Supplement: Supplementary file 1 [file cancers-16-00304-s001.zip › Supplementary Figures.pdf]

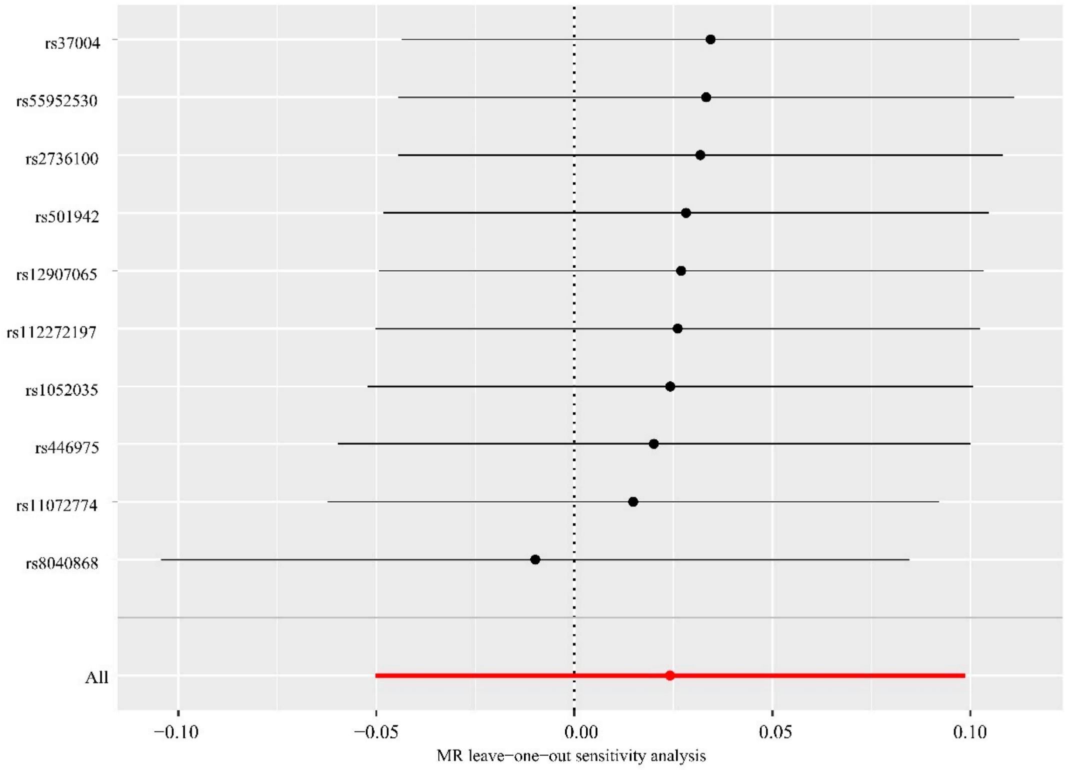

Supplementary Figure S1: The leave one out sensitivity test for lung cancer and hospitalization after COVID-19.

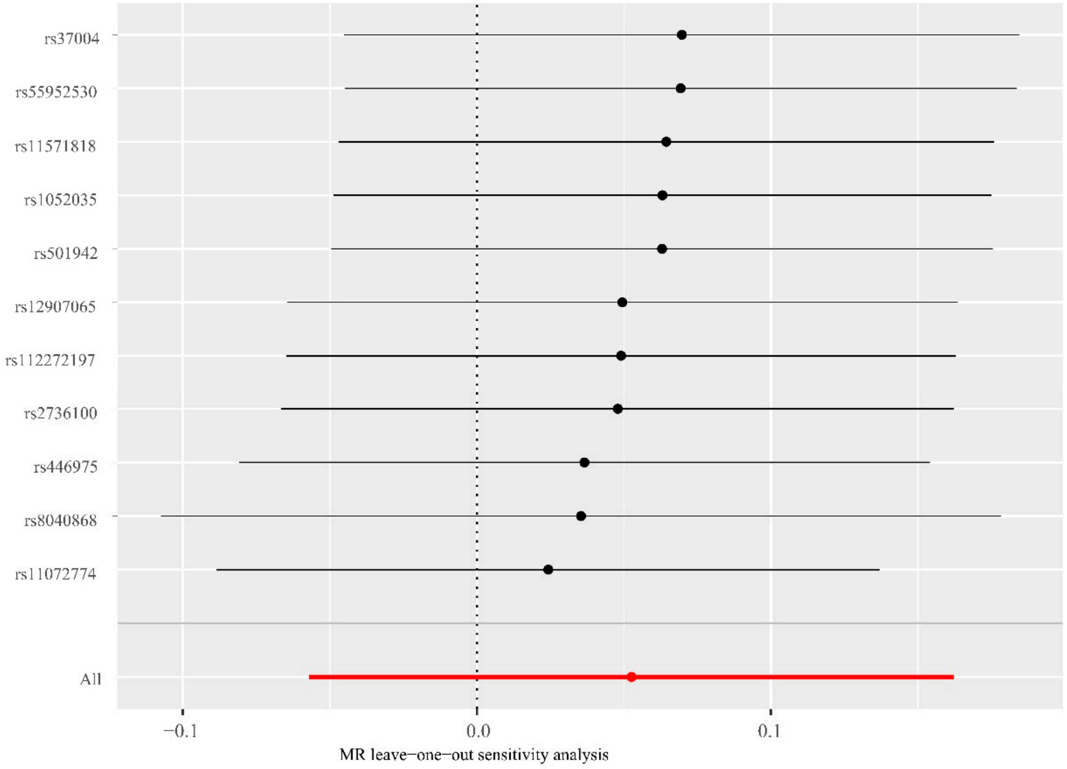

Supplementary Figure S2: The leave one out sensitivity test for lung cancer and severe COVID-19.
